# Concepts as plug & play devices

**DOI:** 10.1098/rstb.2021.0353

**Published:** 2023-02-13

**Authors:** Nicholas Shea

**Affiliations:** ^1^ Faculty of Philosophy, University of Oxford, Radcliffe Humanities, Woodstock Road, Oxford OX2 6GG, UK; ^2^ Institute of Philosophy, University of London School of Advanced Study, Senate House, Malet Street, London WC1E 7HU, UK

**Keywords:** concepts, simulation, compositionality, semantic cognition, grounding, learning-by-thinking

## Abstract

Research on concepts has focused on categorization. Categorization starts with a stimulus. Equally important are episodes that start with a thought. We engage in thinking to draw out new consequences from stored information, or to work out how to act. Each of the concepts out of which thought is constructed provides access to a large body of stored information. Access is not always just a matter of retrieving a stored belief (semantic memory). Often it depends on running a simulation. Simulation allows conceptual thought to draw on information in special-purpose systems, information stored in special-purpose computational dispositions and special-purpose representational structures. While the utility of simulation, prospection or imagination is widely appreciated, the role of concepts in the process is not well understood. This paper turns to cognitive and computational neuroscience for a model of how simulations enable thinkers to reach novel conclusions. Carried over to conceptual thought, the model suggests that concepts are ‘plug & play’ devices. The distinctive power of thought-driven simulation derives from the ability of concepts to plug into two kinds of structure at once: the combinatorial structure of a thought at one end and special-purpose structural representations at the other.

This article is part of the theme issue ‘Concepts in interaction: social engagement and inner experiences’.

## 1. Introduction

Concepts are the recombinable components of conscious, deliberate thought. When Darwin asked himself *shall I marry?* his thought made use of his concept of marriage. Concepts are found in our judgements, hypotheses, intentions, aims and occurrent desires and beliefs. Their subject matter encompasses the concrete and abstract, particulars and properties, the physical, logical and socio-cultural, and runs from the mundane to the extraordinary: dog, number, heavy, and, Monday, bus, Mandela.^1^ They are sub-propositional: a concept does not make a claim about the world on its own, but only when combined with other concepts into a complete thought. Here, only when a representation can figure as a sub-propositional recombinable component of deliberate, conscious thought^2^ will it count as a concept. (Such a representation might also show up elsewhere, e.g. in long-term memory or in non-conscious processing). That stipulation serves to pin down our topic, concept-involving thinking.

Most work on concepts focuses on categorization—understandably, since experiments have been designed so that participants can respond to a stimulus. But just as important is the capacity to use concepts to think through what to do, or to work out what is the case, unprompted by a current incoming stimulus. These episodes of thinking begin with a conceptual thought.

Each concept in a thought affords access to a rich body of information. Some information can be accessed as explicit semantic memories: further conceptually structured thoughts. For example, my concept of Paris allows me to retrieve the belief, *Paris is the capital of France*. But often the way concepts provide access to information is not simply a matter of retrieving stored information. It depends on running simulations (in a broad sense). For example, in prospective reasoning the thinker uses the resources of episodic memory to imagine what a certain scenario would be like. In sensorimotor planning, the thinker simulates an action, observes the likely outcome and evaluates it, in order to decide that to do ([3], pp. 152–160). In spatial planning, the thinker simulates routes through the environment in order to decide which one to follow. A simulation does not simply retrieve a fact stored in memory. But it does allow conceptual thinking to rely on and make use of information found in other systems, information that is stored in special-purpose computational dispositions and special-purpose representational structures.

When theorizing about the way that concept-involving thoughts drive offline processing, the usual paradigm is reasoning. In reasoning we move from some thoughts to others using a general-purpose computational process that is rather like theorem proving in logic. If I want to visit Paris and recall that Paris is the capital of France, then I can reason my way to the conclusion that I ought to polish up my French. Human deductive reasoning can plausibly be modelled in this way. It has proven famously tricky to expand this into an account of ampliative or abductive inference ([4], pp. 115–126). Simulation is a different, complementary, way of performing inferences. It may account for many of the cases that (since they lie beyond the purview of reasoning within a language of thought) Jerry Fodor declared to be the great mystery of cognitive science.

A focus on reasoning has meant that the importance of simulation, prospection or directed imagination has been downplayed. Even Johnson-Laird's rich mental models approach [5] in practice focuses on how thinkers make deductive, inductive and modal inferences. It has little to say about the kind of simulation, prospection and imagination at issue here [6].

At the same time, a smaller stream of work has highlighted the way thinkers arrive at new beliefs on the basis of sensory or sensorimotor emulation or imagination [7,8]. Laurence Barsalou has famously argued that this is what a concept consists in—a simulation of a perceptual state [9,10]. Without endorsing that claim, the rich body of data he and his collaborators have collected does show that sensory, motoric and affective processing has a strong influence on the way conceptual thinking unfolds. Nancy Nersessian relies on these findings to argue that one way in which people solve problems is by constructing a mental model that is manipulated through simulation of events, situations and processes [6]. Theorists have puzzled about whether this is a form of inference or whether it is more like observing the world when performing a real experiment. Plausibly, it is neither: it is its own kind of thing [11]. I use the term ‘thinking’ broadly so as to include processes that reach a conclusion via a simulation.

Running a simulation gives conceptual thinking access to information that could not simply be retrieved from memory or inferred by reasoning. For example, in a classic series of experiments, people were asked questions about tipping glasses of water. They had to predict when water would spill out. Reasoning about the problem usually produced the wrong answer. People who imagined performing the action were able to get the correct answer [12]. Trying out scenarios in imagination has the advantage of not having to suffer the consequences of a real experiment (hypotheses can die in our stead: [13]). Given the opportunity to do both, people can weigh the relative costs and benefits of each [14].

Deliberate thinking uses working memory, so it is no surprise that the kind of mental simulation we are concerned with depends on working memory resources in the prefrontal cortex (PFC). Working memory activates contents in sensory areas. During visual imagination, dorsal PFC influences activity in posterior visual areas ([15], pp. 380–387). The same is true during planning. Planning can also involve motor imagery in pre-motor cortex and parietal cortex ([15], pp. 380–387). Imagining future events (prospection) draws on the episodic memory system in the hippocampus ([15], pp. 390–395; [16]).

It may seem strange that a simulation can allow a thinker to discover something new [8]. Simulation depends on the thinker already having the information available, in some sense. The information may be encapsulated in a special-purpose system and therefore not directly available for use in conceptual thought. Running a simulation is a way of bringing the information into conceptual thinking [11]. A complementary idea is that perceptual systems encode situational constraints ([10], p. 536). These constraints guide a simulation so as to take us to novel conclusions [6,17]. Assumptions about the world can be implicit in the way representations are processed [18]. For example, the visual system is disposed to transition from a certain distribution of contrast and edges to representations of shapes [19]. Those dispositions implicitly encode assumptions about how contrast and edges carry information about shapes, given the statistics of our environment. Processing dispositions are in effect coding implicit assumptions. Those assumptions constrain how a simulation unfolds. A thinker may have little explicit knowledge about the statistics and physics of their environment, but if they simulate actions, their conclusions about what will happen are effectively informed by the information encoded implicitly in the processing dispositions of their sensorimotor systems.

Special-purpose systems need not be modality-specific (visual, auditory, motoric, etc.). Information about a specific domain may be encoded in amodal structures [1,20]. The cognitive map of the spatial environment, encoded in the medial temporal lobe, is a prominent example [21,22]. Similar structures can be used to represent the distribution of properties in other domains, like the leg length and neck length of a set of hypothetical birds [23], or the social hierarchy of a set of individuals [24]. There is evidence that different areas of the brain encode certain domains of objects and properties in a high-dimensional feature space [20,25]. These are all cases of structural representation: a structure over a set of representations serves to represent relations and properties in the world ([26], p. 118). A structural representation is special-purpose in the sense that its representational significance depends on the way it corresponds to the domain it represents. Information represented in a special-purpose structural representation may not be directly accessible to conceptual thought. Running a simulation is a way for concept-driven thinking to rely on, and thus reap the benefit of, that kind of information.

In short, special-purpose systems encode a wealth of modal and amodal information. This may be encoded in structural representations, it may be implicit in processing dispositions, or it may be explicitly represented but encapsulated from conceptual thought. Running a simulation offers a way to derive conclusions that rely on, and therefore make use of, special-purpose information in all these forms.

Although the existence of various forms of simulation is widely recognized, how it works computationally is not well understood, especially in the case of simulations driven by concepts—by concept-involving thinking. Non-human animals engage in various forms of prospection and simulation [15,27]. Tests of mental planning or simulation are a standard non-linguistic way of probing animal intelligence [28]. The capacity for simulation does not require the capacity for conceptual thought in our sense. Simulations may occur within a special-purpose system, for example in route planning [29]. Our question is how simulation works when it is driven by conceptual representations. How do concepts drive simulations or interact with them?

To answer that question, my tactic will be to appeal to a model of how offline use takes place within special-purpose systems, a process that has been discovered by cognitive neuroscience and specified precisely in computational models. The model, in short, is that a representation can be a ‘plug & play device’. Representations that are connected to the environment online, driven by incoming stimuli and driving behaviour, can come to acquire useful properties—through various mechanisms of plasticity or learning they form interconnected processing dispositions, or they become connected into a representational structure. If those representations can be taken offline and ‘played with’ in a simulation, the simulation can make use of the representational structures and implicit information that were built up online. Representations are unplugged from the world and plugged into a simulation. Playing with representations in an offline simulation allows conclusions to be derived from the information encoded, in various forms, in special-purpose systems.

The argument of this paper is that a concept is also a plug and play device. But there is an important difference between concepts and the plug and play devices found within special-purpose systems. Concepts are also plugged into the compositional structure of a thought (see §6). They are effectively plugged into structures, of different kinds, ‘at both ends’. That makes a crucial difference to what conceptual thinking can achieve.

## 2. A model of offline processing in special-purpose systems

This section summarizes work in cognitive neuroscience and computational modelling on offline use and replay of representations in special-purpose systems. This offers a precise characterization of what it is for a representation to be a plug & play device. In §3, that idea is applied, with an important modification, to concept-driven thinking.

Our first example is spatial navigation, as supported by representations in the hippocampus and wider medial temporal lobe. This is studied most intensively in rodents, with converging evidence that the same process is found in humans [21,22,29,30]. The computations take place subpersonally, within a psychological subsystem. They are not thought to involve personal-level reasoning, although their output does feed into decisions taken by the whole agent. The medial temporal lobe harbours various representations that interact to form a cognitive map of an animal's spatial environment. Components include place cells, grid cells and head direction cells [31]. For simplicity, I focus here on place cells and a somewhat stylized description of the way place cells are involved in calculating the shortest route through a landscape.

Each place cell is active at a specific location in a given spatial arena. One cell might respond when the animal is in one corner, another when it is halfway down one of the walls. The location sensitivity of a place cell is a rich computational achievement in its own right. A place cell can be activated by complex visual cues, invariant to gaze direction. It can register location non-visually through local smells and olfactory gradients. Place cell activity is integrated with head direction cells and proprioceptive feedback from limb movement so that location information is updated by the animal's own movement, even in the dark. Plausibly, a place cell can also act as a target, directing activity that takes the animal to the corresponding location. Thus, each place cell is at the apex of a sophisticated package of sensorimotor processing giving it highly specific input- and output-selectivity.

As a result of experience, a structure is built up over the array of place cells. Cells that correspond to nearby locations get wired together so that, subsequently, they tend to activate one another. The relation of co-activation between place cells thereby comes to mirror the relation of proximity between locations ([26], pp. 113–6). Route calculation depends crucially on being able to sever, temporarily, the array of place cells from their input- and output-sensitivity. Only the relational information encoded by the co-activation relation is used in route planning. The system can run through different routes offline in order to select the shortest. Having relied on these internal relations to compare the length of different potential routes, and having selected one, sensitivity to the environment is restored so that place cells can be used to guide action.

Having representations with the kind of sophisticated input- and output-sensitivity exemplified by place cells is obviously a useful resource. But so too is the ability to disengage from these robust correlations with the world—to take the array of place cells offline. Computations made with the array of representations while they are offline rely on the structures that have been built up over those representations online. Running a simulation with representations offline enables computational use to be made of the information carried by those structures.

This example delivers a useful insight—a computational principle that can be deployed in many places. It shows how a representation can be a plug & play device. Useful as it is to have representations that correlate in a sensitive and specific way with conditions in the environment, it is even more useful to be able to sever representations from their worldly correlations, run internal computations that make use of their interconnections, and then restore their input–output profile so that the conclusions that have been worked out offline can be used to guide behaviour.

There is evidence that relational structures in the medial temporal lobe, similar to those used to represent spatial locations, are used to represent other kinds of relational structure. One example is the relations between leg length and neck length of a collection of hypothetical birds that participants were trained to manipulate [23]. In another example, a cognitive map of social properties was used to infer social relationships between individuals, also using a grid-like code [24]. The inferred relationships were not encountered during training, and go beyond chains of associations [32,33]. A grid-like code encoding objects or events in a feature space is also found in other brain areas, including the medial PFC [34,35].

Offline simulation in these systems has been found to play a role in consolidating the information that has been learned [36]. In the case of the spatial cognitive map, offline replay during rest or sleep is thought to be involved in consolidating memory of the spatial relations between locations [37,38]. In the domain of foodstuffs, Barron *et al.* [39] trained people to associate a variety of foodstuffs (e.g. jelly; tea) with neutral stimuli. They found that activation of these memories was involved in constructing a representation of a novel foodstuff (e.g. tea jelly). Imagination is a way of simulating new possibilities within a domain, allowing new connections to be formed or novel categories to be represented [35,40].

Another area where offline use of representations is obviously crucial is in ‘model-based’ planning. A model-based system has a representation of states of the environment, how they are connected, and how actions that the agent can perform will move it between states (e.g. turning left at L1 takes you to L2; pulling a lever when the light goes on delivers sugar solution). In working out what to do, a computation runs through various possible chains of states and actions in order to evaluate which action in the current state is likely to produce the largest long-run reward. Sometimes the world model is programmed into the system at the outset, for example by telling the agent the possible board positions and legal moves of a game like chess. Other models are more like the spatial map in the hippocampus in that the structure of the environment is learnt from experience [41]. In both cases, representations are manipulated offline to reach a decision about what to do. Furthermore, even when not faced with an immediate choice to make, it seems that simulations performed in the model-based system train up the model-free action policy that is relied on when faced with an immediate decision [42].

Computational models show how to resolve the apparent paradox mentioned at the outset—that simulation only allows the thinker to learn something if in some sense they knew it already [11]. Information encoded in a dispositional connection between stimuli (like those in the [39] study) cannot itself act as an input to an inferential process (cp. Halford *et al*. ([43], p. 499), ‘An associative link *per se* cannot be an argument to another association’). Running simulations in a model of the environment (background planning) allows the system to reach conclusions about how best to act in various circumstances, conclusions that rely on the constraints encoded in the model [44]. Even apart from modelling human cognition, computer scientists have found that simulations in the form of ‘experience replays’ are useful in their own right as a tool for training deep neural networks [45].

These findings from cognitive neuroscience and computer science offer a template for the way offline simulation can extract information from a special-purpose system: not simply by retrieving a stored representation, but by relying on special-purpose computational dispositions and special-purpose representational structures. Where a representation is a plug & play device, simulations run with it offline can make use of information built up online as a result of experience. This is the model that I want to carry over to cases where simulations are driven by concept-involving thoughts.

## 3. Concepts as plug and play devices

We have seen special-purpose ‘plug & play’ representations at work in cognitive neuroscience and computer science. These discoveries offer us an insight to apply to understanding the cognitive–computational mechanisms underlying the mental simulations driven by conceptual thought. The claim of this paper is that concepts, too, are plug & play devices. They give us a general-purpose ability to make offline use of representations—not just offline use of a concept itself, but offline use of the special-purpose amodal, sensory, motoric, affective and evaluative representations to which a concept is connected.

When a conceptual thought prompts an episode of mental simulation, special-purpose representations are activated, not driven by current perceptual input, but driven by the episode of thinking. The thought of *soldiers mounted on polar bears* prompts certain sensory imagery. By running a sensory simulation, I imagine what the scenario might look like and I predict the likely consequences (including for the hapless soldiers). As we saw with route planning, it is extremely helpful to be able to take special-purpose representations offline to derive consequences that follow from the information encoded, explicitly or implicitly, in the special-purpose system. Conceptual thought seems to give us the capacity to do that across the board. A concept is typically connected to a rich body of information about its subject matter: sights, sounds and smells, emotional resonances, sensorimotor affordances, evaluative appraisals and amodal semantic relationships. Conceptual thought gives us the capacity to take all or most of that material offline and run simulations with it. Holding a conceptually structured thought in working memory is a general-purpose means for making offline use of special-purpose bodies of relevant information.

Our ability to do that is so familiar that it is easy to overlook just how useful it is. Like all animals, humans are equipped with a variety of specialized psychological mechanisms: to perceive the environment and many kinds of things in it, to learn about rewards and harms, to coordinate action with the complexity of the environment and the organism's internal needs. Each of these sophisticated special-purpose computational capacities encodes, explicitly or implicitly, a store of useful information. Having the generalized capacity to run simulations in any of these systems would be very useful indeed. Concepts seem to give us that capacity. Not only does conceptual thought allow us to take all or most of our special-purpose systems offline. Concepts also give us a way to focus on and manipulate any of the objects, properties or relations that are represented in those systems. That can be a matter forming thoughts and simulations of situations we have already encountered. It can also involve recombining concepts so as to simulate novel scenarios.

The combinatorial power of conceptual thought has long been recognized. Putting together existing concepts allows us to think new thoughts. Less remarked on is the fact that this normally brings with it the capacity to simulate or imagine a scenario corresponding to the novel thought. I can formulate the thought *a cart that runs on an iron road* and then start to imagine what that would look like and how it would work (a railway). When I suggest *a regiment of soldiers mounted on polar bears*, you will start to imagine what that will look like and what might happen. There is considerable evidence that thinking with concepts activates representations in multiple special-purpose systems—sensory, motoric, affective, evaluative and amodal [9,10,23,33,46,47]. The power of conceptual recombination lies not simply in the capacity to recombine labels or words in a language of thought. Its power lies in the capacity to simulate scenarios corresponding to our thoughts.

Thus, structured conceptual thought can allow us to simulate a scenario that we have never encountered before and to work out possibilities for action. Having read a tip in an online climbing forum, I can now imagine using a toothbrush attached to a telescopic pole to clean bird droppings off a ledge high up on a cliff. The simulation leads me to realise that the brush needs to stick out obliquely from the pole (and to watch out for dirt falling in your eyes). As we have seen, each special-purpose system contains its own store of useful information: what a tool will look like from different angles; how a fruit will smell when cut open or how it will feel when crushed; general visuo-motor affordances for action; which rewarding outcomes are available in different situations, and which dangers; what emotional experiences are likely to be involved; and how objects and their properties are systematically related (in an amodal semantic space). The special-purpose information has been built up as a result of experience. It is deployed online as we are moving through the world, perceiving and acting. Simulations take those capacities and run them offline. Recombining concepts into a new thought allows us to put those capacities together in novel ways. The idea that a concept is a plug & play device serves to draw attention to the fact that this remarkable ability is something more than the capacity to recombine concepts in thought. We can build a suppositional scenario equal to the thought.

Consider an example of how conceptual thought allows for recombination across different special-purpose systems. I'm wandering round the local farmers' market trying to a plan a meal I will cook for friends that night. A special offer on sea bass gives me some ideas. I picture some of the other products I have seen and imagine them put together into a dish. Each simulation prompts an evaluative response. Sea bass with those lovely-looking squash? Euch. Sea bass with those fresh-looking bulbs of fennel? Maybe. Perhaps roast the fennel. What about pulses? Oh yes, chick peas on that organic stall. And some hard-crust sourdough bread. And so on. I'm using my spatial map of the market, sensorimotor knowledge of how to process raw ingredients, olfactory and gustatory simulation of potential dishes, and perhaps knowledge of a state space interrelating potential flavours. Concepts allow us to manipulate specific components of the scenario: change chick peas for puy lentils, change the colour of the carrots from orange to purple, fry the fennel instead of roasting it. Evaluations are brought to bear at every step. Will the meal I am imagining taste good? Is it too far to walk back to the bread stall? Conceptual recombination allows me to form thoughts of various novel dishes but, as I stand next to the fish stall staring into space, it is the capacity for simulation that allows me to consider what the dishes will be like, how to prepare them, and how to forage for the various ingredients.

In short, concepts give us a general-purpose way of taking special-purpose systems offline so as to run simulations. They allow us to manipulate specific components and to recombine those components in novel ways.

## 4. Types of information connected to a concept

Examples of simulation tend to focus on modality-specific representations (visual, auditory, tactile, olfactory, etc.). However, simulations can equally involve amodal representations. My hypothesis is neutral between so-called ‘grounded’ and amodal theories of concept-driven simulation [1]. I am understanding simulation broadly so as to cover, as well as imagination and prospection, model-based suppositional thinking: deploying a model of the environment offline so as to consider what would happen in various hypothetical scenarios.^3^ The domain-specific or task-specific representations involved in suppositional scenarios can be amodal [1,20,48]. Where *modal* representations are relied on, these need not be as detailed as perceptual experiences (representations driven by current input). Rather than being like a photograph, they may abstract in various ways from online representations.

Activating a concept serves to rapidly activate an extensive body of associated information, some of it only transiently [49]. We can think of the concept as ‘pointing’ to a body of information stored in memory [50]. The information accessed by or through a concept encompasses sensorimotor representations, including affordances for action and embodied experiences [46]. However, our knowledge about objects and their properties also appears to be organized into amodal domains of semantically related entities, domains such as the social, emotional, mental, professional, violent, numeric and temporal, as well as the tactile and visual [25,51]. And even modality-specific representations may be relied on in a simulation for their more abstract structure. Analogical reasoning depends on perceptual and kinaesthetic representations, not just amodal information [52,53]. This seems to involve using a perceptual relation to stand in for another relation [54]. Liz Camp has introduced the term ‘characterizations’ to encompass the many types of information, in addition to explicitly represented semantic beliefs, that can be encoded by or with a concept [55]. The power of concept-driven simulations is that they can draw on any of the characterizations connected to a concept.

The representations in an unfolding simulation need not be conscious. During the process of deliberately deciding which way to set off from the library at the end of the day, I may not be consciously aware of the route planning going on in my hippocampal cognitive map. I may just be aware of the result: *set off this way*. Aspects of a deliberate thought process do figure in the stream of consciousness: prominently, concept-involving thoughts themselves (occurrent desires and beliefs, goals, intentions, judgements, etc.) and often also domain-specific states (the imagined sight, smell and taste of a potential meal, together with my affective assessment of it). But simulation can also proceed via domain-specific representations and structures that remain unconscious.

## 5. General-purpose combinatorial structure

Concepts combine into thoughts using a highly general mode of combination (the ‘generality constraint’: [56]). To a first approximation, any concept can combine in thought with any other. More carefully, it appears that the ability to combine two or more concepts into a complete thought is not in general restricted by the particular subject matter that the concepts refer to (e.g., to take concepts from several of the domains discovered by [25]: *a violent introvert five-foot-tall pianist*). If there are certain thoughts that we cannot think, that is not because the concepts are prevented from combining because of the way they are represented. Contrast representations in the cognitive map of space. The spatial cognitive map represents relations between spatial locations and is restricted to representing spatial relations. It cannot represent any arbitrary relation between locations (e.g. relative rental cost per square foot). Or consider binding in the visual system. There are only certain properties that can be represented as attributes of a visually tracked object. Colour and shape can be bound to a visual object, but probably not personality type. Recombination of concepts, by contrast, does not seem to be restricted by subject matter. (Whether we will succeed in simulating or imagining a scenario equal to the thought is another matter.)

The way concepts combine in thought is similar to the way words combine in language. This could be because conceptual thinking makes use of the language faculty, perhaps involving sub-vocalized words or auditory imagery. It could be the other way around: that the compositional structure of language reflects the compositional structure of thought. Or these could be two separate but related capacities, with some conceptual thought drawing on the language system and other conceptual thought carried out in a combinatorial representational system that is independent of the language faculty. My hypothesis about conceptual combinatorial structure can remain neutral on this issue.

The capacity to combine concepts depends on representations in prefrontal cortex [20,43,57]. A popular account is that prefrontal cortex sets up temporary task-relevant representations in working memory. We can think of these as neutral labels or pointers connected to richer representations in special-purpose systems, for example in anterior temporal cortex [20]. The representational structure in prefrontal cortex that interconnects working memory labels is not tailored to any particular domain or subject matter. It is language-like in that it employs ‘combinatorial procedures that are distinct from the contents over which they operate’ ([20], p. 295). The richer representations connected to the labels have more special-purpose structures, for example map-type structures, or the structure of a particular perceptual modality. When people reason by drawing explicit analogies, they do so by setting up a mapping amongst neutral working memory labels [43,57]. When running a simulation, the working memory labels serve to activate, sustain and manipulate domain-specific contents. Since the working memory labels can be combined in arbitrary ways, there is no in-principle limitation to the thoughts that can be entertained.

Studies of category learning support this distinction between two different levels of processing [58]. One level is multi-dimensional, implicit and automatic, the other low-dimensional, rule-based and deliberate. Category learning in the deliberate system is a matter of inferring a rule based on one or two features of the stimuli, e.g. that Xs have long necks and no spots. The category label may be given before or even long after the samples to be categorized, and feedback about correct and incorrect classification is not essential. On the other hand, performance is impaired by cognitive load, by having to perform a second concurrent task (a symptom of the ‘type 2’ or ‘system 2’ style of processing). Automatic category learning by contrast shows minimal interference from a dual task. It can learn a category demarcated by a large number of features in a high-dimensional state space. Learning is better if the category label comes after the samples. Feedback on performance is essential and the response must be made within a short time window of the stimulus. Learning is impaired by simply switching the location of the response key.

My suggestion is that these two systems draw on opposite ‘ends’ of the plug & play device that is a concept. The deliberate system makes use of reasoning on syntactic structures, with conceptual labels embedded in a compositionally structured thought. It draws on explicit conceptual representations of what it takes for a sample to fall under the concept. The automatic system operates on the material at the other end. It uses a more implicit, feedback-based learning mechanism to carve out a region of high-dimensional feature space corresponding to the category. Since concepts are keyed into both systems, categorization judgements can draw on either kind of information, depending on the nature of the task (e.g. in a context where there is cognitive load, that will impede the type 2 system).

## 6. How do simulations come to reflect combinatorial structure?

**Figure 1.**
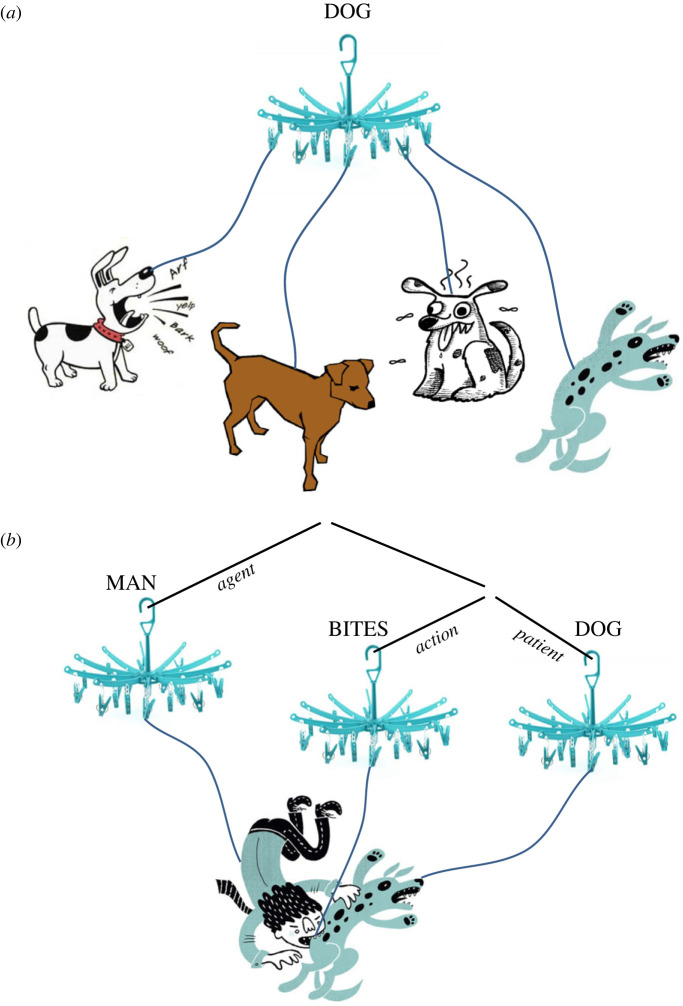
(*a*) A label in working memory is connected to many different representations in special-purpose systems. (*b*) Working memory labels combine to form a thought. The thought drives a simulation of an integrated scenario that reflects the mode of combination of the labels. (Images from http://clipart-library.com. Graphic copyright the author). (Online version in colour)

So far we have seen that a concept is plausibly a plug & play device. Like plug & play representations within a special-purpose system, a concept allows representations to be taken offline and deployed in a simulation. The simulation draws on dispositions and structures that have been built up online. However, concepts are also importantly different. If a concept is a plug & play device, it is a device that plugs into structures at both ends. At one end there is the structure of representations in a special-purpose system. At the other end, it is plugged into the combinatorial structure of conceptual thought. Each concept is like a hook with a collection of odd-shaped objects hanging off it. The hooks can be hung up together any way you like, but it is a substantial achievement to get the collection of dangling odd-shaped objects to arrange themselves into a coherent picture (figure 1).

Jackson *et al*. [2] argue that concept-driven thinking (controlled semantic cognition) has to perform two functions at once that pull in opposite directions. It needs to deploy and combine context-invariant conceptual representations, with contents that are not tailored to a specific time, place or modality. But it also needs to connect up conceptual thought to modality-specific perception and behaviour in a way that is suited to a particular context and task. Using neutral labels or pointers enables context-invariance. Each label acts as an unstructured representational constituent, either because there is no structure within a concept ([59], pp. 90–93; [50]), or because any structure that is present has no effect on the processes of combination and reasoning that take place over labels. But that leaves the other half of the problem unresolved: how to achieve context-sensitivity.

Specifically, the information recruited into a simulation by a concept has to be sensitive to the other concepts with which it is combined. The simulated content driven by apple in the context of apple tree will be very different from that recruited by apple in the context of apple crumble. ‘A full understanding of a particular combination must flexibly estimate the interactions between the component parts' ([20], p. 17.9). Special-purpose resources will carry some of the load here, selecting a collection of components that fit together with one another—that make sense within the representations and structures of the special-purpose system. Simulating green leaf will select for shades of green consistent with natural foliage; green sea selects for different shades.

However, a deeper problem presses. A simulation has to respect the mode of combination over the labels. We imagine something different for dog bites man than for the rather more surprising man bites dog. A thought is not just a list of concepts. A simulation has to do more than simply construct a scenario consistent with the collection of contents being entertained. The way that special-purpose resources are combined in a simulation has to somehow reflect the combinatorial structure of the corresponding thought.

Models of conceptual combination tend to focus on one aspect of Jackson *et al*.'s problem and overlook the other. In one model, the pointers or labels are addresses for the location of stored contents [60]. In processing a three-word sentence, the PFC has separate memory stores for agent, verb and patient. Each contains the address where the relevant content is stored. This does not tell us why something different is simulated when ‘dog’ is the agent of the verb ‘bite’ rather than the patient. Frankland & Greene [61] found that the identity of agent and patient are coded in distinct subregions of left mid superior temporal cortex, one for dog-as-patient and another for dog-as-agent. That would achieve context-sensitivity, but does not explain invariance, the invariant content of dog shared between the two roles. Frankland & Greene [61] suggest that both patterns of activity are in turn pointers to shared content, which is housed elsewhere in cortex (p. 11737). But that does not explain how the role-specific content is based on or constructed from the context-invariant content.

Another model uses content-addressable memory. For example, in the semantic pointer architecture of Eliasmith [62], the representations that enter into combinations are compressions of richer perceptual representations. They are not neutral labels, but pointers to richer contents. Combinatorial operations are defined on the pointers that are reversible, allowing the components to be recovered (the pointer is ‘dereferenced’). This is like the tensor product architecture suggested by Smolensky [63]. A vector for *dog* is convolved with a vector for *agent* so as to form a representation of *dog-as-agent*. The identity of the agent (dog) can be extracted from the convolution and decompressed. Blouw *et al*. [64] use circular convolution for vector combination, which can be applied recursively (although the components, since they are compressed, are only imperfectly recoverable). Halford *et al*. [43] use tensor products, so that the representation for *Sally loves John*, say, is the tensor product of three vectors, one for Sally, one for John, and one for the relation Loves(x,y). The differentiable neural computer architecture of Graves *et al*. [65] achieves the same effect by separating operations performed by a ‘controller’ from the contents being processed (vectors retrieved from memory). The hybrid symbolic-connectionist architecture of LISA has dedicated individual units for each object and each relational role [66]. These models all successfully implement context-invariance of conceptual contents across different modes of combination. What they do not explain is how those contents are systematically modulated according to the mode of combination: why the dog that is simulated for *dog bites man* is different from the dog that is simulated for *man bites dog*. Indeed, these models all use combinatorial principles, like taking tensor products with role vectors, designed so that the way dog will be decodable from dog-as-agent, say, is not affected by the content found in the patient position.

How, then, to implement combinatorial principles that do not depend on the contents of the components being combined, while ensuring that simulations driven by a conceptual thought reflect the combinatorial structure? Here is one potential model (following suggestions in [20]). We have seen that the rich contents connected to a concept (like dog) can be coded in a high-dimensional state space in a special-purpose system (amodal or modal). The state space encompasses many features over which items in that domain can vary (e.g. features of animals). Particular concepts (e.g. dog) correspond to regions in the space. A particular instance is represented by a point in state space (a particular dog). One way of adjusting to context is to move between regions of state space. For example, in the context of big, the concept dog will only activate regions of its state space for which the size feature is relatively large (relative to the range of sizes that fall within the dog region). To take another example, green leaf will encompass one range of values along the colour dimension, green sea will encompass another.

My tentative suggestion is that there are also dimensions in special-purpose state space that correspond to compositional roles. For example, there is a dimension in the dog state space that corresponds to the agent–patient distinction. When working memory labels are combined into the structure *dog-bites-man*, the label for dog is assigned *agent* as a feature. The contextually appropriate simulated scenario then has to make use of the subregion of the dog state space that corresponds to *agent* on the *agent–patient* dimension. In effect, when so-combined, the label for dog serves to activate only information consistent with dog-as-agent. Further interactions with the regions connected to the other labels (man, biting) result in the construction, in special-purpose state space, of an integrated scenario where the regions of state space activated by each label are adjusted to fit with one another.

According to this suggestion, the syntactic structure into which working memory labels are combined furnishes semantic constraints on the types of scenario that will be simulated. In fact, much of the work in linguistics on compositional semantics is focused on pinning down these constraints (in the case of language comprehension). Syntax constrains the type of scenario with which a thought can be fleshed out. Jerry Fodor's classic view is that there is a language module that parses linguistic input, outputting a linguistic form, which is just a structured representation of lexical items, with no semantic analysis ‘inside’ lexical items ([59], pp. 90–93). More recently, Paul Pietroski has published a sophisticated theory in which word meanings are underspecified. Syntactic structure tells the comprehension system what kinds of information to look up, for each word, in order to understand a sentence [67].^4^ Another model is that the syntactic and closed-class elements of a sentence specify semantic content, but only of a very general kind. For example, the closed class elements in the sentence ‘Those boys are painting my railings' specifies that the meaning has to be a scenario in which *those somethings are somethinging my somethings* [68]. These are all ways in which syntactic structure can serve to constrain the semantic possibility space within which a scenario will be simulated.

My suggestion, then, is that the compositional structure of a conceptual thought constrains which subregions of high-dimensional feature space are made available by each concept. The simulated scenario is constructed out of the interaction of these subregions. This is just a sketch. Much more is needed to specify a detailed model, properly informed by empirical findings. The sketch is useful in so far as it highlights the nature of the problem.

The hypothesis that concepts are plug & play devices is plausible. It is supported by empirical findings and computational modelling. The dog-bites-man / man-bites-dog question then becomes pressing. More research is needed to discover how thought-driven simulations are constrained to reflect the combinatorial structure of thought.

## 7. Conclusion

Concepts provide us with a way of running offline simulations in any of our special-purpose systems. A concept allows us to manipulate specific components of a simulation, for example the colour of an object in an imagined scenario. Concepts combine in a general-purpose way, which allows us to form novel recombinations, and to bring together special-purpose systems in a simulation that transcends our real-world experience.

This convergence of general-purpose and special-purpose features makes concept-driven thinking especially effective. It allows us to draw on the information that has been built up within special-purpose systems as a result of experience. Simulation takes place in working memory. Representations are maintained and manipulated, constructing an integrated picture of a situation or hypothetical possibility. Those are the functional features of the global workspace [3]—of representations that are conscious [69]. The information contributed by each constituent concept is selected so as to mesh with the others in the workspace, and to reflect their mode of combination. So it seems that consciousness is crucial to this mode of thinking. Conscious, deliberate, conceptual thought unites (i) a general-purpose way of taking information offline and recombining elements, with (ii) a context-sensitive way of integrating special-purpose systems so as to represent integrated scenarios in a global workspace. That is possible because each concept is a plug & play device. Therein lies the special power of thinking with concepts.

## Data Availability

This article has no additional data.

## Footnotes

1 Small caps refer to concepts.

2 Cp. ‘endogenous control’ [1]; ‘controlled semantic cognition’ [2].

3 These are often called counterfactuals, but their suppositions needn't run counter to the facts. They are scenarios we would capture with subjunctive conditionals, whether or not the antecedent turns out to be false, e.g. if it were to rain, xyz would happen.

4 In Pietroski's terminology, word meanings are instructions for how to access a concept. Which concept is fetched depends on the syntactic structure and grammatical / closed class content of the sentence.
